# Diagnosing tick-borne encephalitis: a re-evaluation of notified cases

**DOI:** 10.1007/s10096-017-3139-9

**Published:** 2017-11-29

**Authors:** Malin Veje, Marie Studahl, Maja Johansson, Patrik Johansson, Peter Nolskog, Tomas Bergström

**Affiliations:** 1 Department of Infectious Diseases, Institute of Biomedicine, Sahlgrenska Academy, University of Gothenburg, Gothenburg, Sweden; 2 Department for Communicable Disease Control in Western Gotaland, Skövde, Sweden

## Abstract

We set out to investigate the serological response of TBE virus (TBEV)-specific IgM and IgG antibodies in stored serum and cerebrospinal fluid (CSF) in notified TBE patients, in order to confirm or reject the diagnosis. We applied the ELISA methods used in clinical practice, Enzygnost and Immunozym, and assessed RT-PCR as a diagnostic tool. A total of 173 TBE cases were notified to the Public Health Agency. Samples from 129 patients were eligible for the study. Stored serum samples were found for 111 patients and CSF samples for 88 patients. All serum samples were analyzed with both Enzygnost and Immunozym, as well as an additional 140 control samples. CSF samples, including samples from ten controls, were analyzed with Immunozym. RT-PCR for TBEV was performed on 126 serum, two whole blood, 96 CSF, two feces and four nasopharynx samples. Only two of 111 notified patients lacked detectable TBEV IgM in serum, from whom one sample was RT-PCR positive. According to the ECDC definition, 117/129 (90.7%) of the reported TBE cases were confirmed. Positive RT-PCR results were obtained in eight patients, one from whole blood and eight from serum samples. Four out of eight of the RT-PCR positive patients were TBEV-IgM positive and none had detectable TBEV-specific IgG. All of the tested CSF, feces and nasopharynx samples were RT-PCR-negative. TBEV-specific IgG was detected in 88.4% and IgM in 31.6% of the CSF samples. RT-PCR on serum samples and CSF IgG antibodies can be used as complementary methods in TBE diagnostics, not least early in the disease course.

**Electronic supplementary material:**

The online version of this article (10.1007/s10096-017-3139-9) contains supplementary material, which is available to authorized users.

## Introduction

Tick-borne encephalitis (TBE) is a zoonosis caused by the flavivirus TBE virus (TBEV), which is mainly spread by tick bites. The disease has a variable severity, ranging from asymptomatic to a critical, sometimes even deadly meningoencephalitis. An estimated 10,000–15,000 cases are diagnosed yearly on the Eurasian continent [1]. In Sweden, the first TBE cases were identified in 1954 [2] and the infection since then has had a steady increase in incidence and affected areas. TBE has been a notifiable disease in Sweden since July 1st 2004 [3], but there has been no published evaluation or quality control of the diagnostic basis for these reports. An accurate diagnostic reporting of notifiable diseases is of great importance for differential diagnostics and epidemiological surveillance, which in turn forms the basis for understanding the mechanisms of spread of TBEV.

Typically, infection with the European TBEV manifests itself as a biphasic disease [4], starting with fever, myalgia and general malaise, followed by approximately 1 week without symptoms, before onset of a variety of neurological symptoms [5].

There are three genetic subtypes of TBEV, TBE-Sib (Siberian), TBE-FE (Far Eastern) and TBE-Eu (European), but only TBE-Eu is known to cause disease in Sweden [6]. Vaccination against one subtype can protect against another [7]. In Sweden TBE vaccination is voluntary.

The TBE diagnosis relies both on clinical and laboratory findings. The clinical picture is wide-ranging, making laboratory testing crucial [8]. In almost all cases, IgM and often IgG in serum can be detected after onset of neurological symptoms. TBEV antibody testing in the cerebrospinal fluid (CSF) is considered a reliable diagnostic tool. CSF antibodies are found in a majority of the patients and develop during the first weeks after onset of neurological disease [9]. TBEV-specific IgM and/or IgG were found in 84/100 patients at the time of admission, in 100% of the patients within 15 days in a study by Kaiser et al. [10], and in 35/36 (97%) on days 7–19 after onset of meningoencephalitis in a prospective Swedish study, where patients with predominantly encephalitis symptoms had significantly lower intrathecal antibody concentrations compared to patients with milder symptoms [9].

According to the definitions of the European Center for Disease Control (ECDC) Meeting Report in 2011, a confirmed TBE case requires both clinical symptoms of TBE *and* IgM plus IgG in serum *or* IgM in the CSF *or* IgM plus IgG in the CSF *or* detection of TBE viral nucleic acid in clinical specimen [11]. Prior to the ECDC meeting in 2011, there was no European consensus regarding the definition of a TBE case. In Sweden, two different commercial ELISA methods are commonly used, Enzygnost (Siemens) and Immunozym (Progen). There is a well-known risk of cross-reactivity between the different flaviviruses (eg. West Nile Virus (WNV), Japanese B Encephalitis virus (JBEV), Dengue virus, and Yellow fever virus (YFV)), which can cause diagnostic difficulties among travelers or vaccinated persons, requiring a neutralization antibody testing in the acute and convalescence stage.

Vaccine breakthroughs are rare but occur, mainly in persons above the age of 50 years. In those cases, a rapid IgG-response is detected whereas the IgM-response may be delayed. To diagnose a vaccine breakthrough poses special problems, where a rise in IgG titer in serum or detection of intrathecal antibodies are needed [5, 12].

TBEV RNA can be detected with reverse transcriptase-PCR (RT-PCR) in serum and whole blood early in the disease, and in some cases in CSF and in urine [13–16]. Previous studies have shown that viral RNA only rarely can be detected in body fluids at the time of onset of neurological symptoms, unless the sample is drawn very early in the disease [14, 15]. At these early stages, serological responses may be weak or absent [15], which is why the diagnostic role for RT-PCR needs further evaluation.

The aims of the present study were (1) to test the quality of the reporting system by investigating the serological response of TBEV-specific IgM and IgG antibodies in stored serum and cerebrospinal fluid (CSF) samples in patients notified with TBE in Western Gotaland, Sweden, during 2004-2012, in order to confirm or reject the diagnosis, (2) to assess RT-PCR as a diagnostic tool for TBEV infection in these notified cases, and (3) to compare the compiled data with the ECDC criteria for TBE diagnosis. 

## Material and methods

### Material

During the study period between July 1st 2004 and December 31st 2012, 173 cases of TBE in the region of Western Gotaland were reported to the Public Health Agency of Sweden, based on clinical symptoms of TBE and on detection of TBEV-specific IgM (Enzygnost anti-TBE virus (IgM), Siemens) in serum. The inclusion criteria in the study were a reported TBE case during the study period and enough saved serum and/or CSF sample material allowing for virological analyses. Samples from 134 patients were retrieved from our regional laboratory and, of these, samples from 129 patients contained sufficient material to be selected for the study. One or more serum samples (*n* = 1–5) were available in 111/129 patients. CSF samples were found in 88/129 patients, of which 20 lacked a paired serum. The majority of both the serum and CSF samples were collected from the first month after onset of the disease. Of the TBE patients, 45% were women and 55% men, with a median age of 47 years (range 4–82). Symptoms, duration of illness, clinical outcome and vaccination status were unknown since we had no access to medical records. Two hundred forty-eight serum samples from 129 patients were analyzed for TBEV-specific IgM and IgG antibodies with Enzygnost (Siemens) and Immunozym (Progen). Sera from 140 age- and gender-matched blood donors were also analyzed, as well as an additional ten CSF samples from healthy individuals from a previous study [17]. Since the blood donors were anonymous, their vaccination status was not specified. Among the 140 blood donors controls, 46% were women and 54% men, with a median age of 47 years (range 18–68).

For RT-PCR testing, samples from two weeks before and four weeks after clinical diagnosis were identified and the first serum and CSF sample from each patient was selected. In total, 230 samples from serum (*n* = 126), CSF (*n* = 96), whole blood (*n* = 2), feces (*n* = 2) and nasopharynx (*n* = 4) were tested with RT-PCR for TBEV. With permission from the Regional Ethical Committee (EPN 999-14), letters were sent to the patients with positive PCR results, and after written informed consent, their medical records were studied. All of the samples were stored frozen at −20 °C and thawed before use.

### Methods

#### ELISA

The Immunozym FSME IgM and IgG tests from Progen (Nordic Diagnostica, Gothenburg, Sweden) and the Enzygnost anti-TBE Virus (IgG, IgM) from Siemens (Siemens Healthcare AB, Upplands Väsby, Sweden) were used to detect TBEV IgM and IgG antibodies in serum, according to the manufacturers' instructions. In addition, the Immunozym test is validated and was used for IgM and IgG analyses of the CSF samples, with dilutions according to the instructions from the manufacturer.

#### RT-PCR

Patient samples from serum, whole blood, CSF, feces and nasopharyngeal secretion were identified. The samples (250 μl of each, and the same amount of feces suspensions) were defrosted, added to 2 ml lysis buffer and incubated at 20 °C for 10 min. Thereafter, RNA extraction was performed by the EasyMag system. Thus, 50 μl of silica magnets were added and the test tube was moderately vortexed. For extraction of RNA from all the samples, the EasyMag feces 250 U1 programme was used, resulting in 110 μl of extracted material. All RNA extractions were carried out successfully and according to the instructions provided by the manufacturer (Biomérieux Marcy-l’Étoile, Gothenburg, Sweden). The extracted RNA was quantified by TaqMan Real Time RT-PCR, as described in 2008 by Brinkley et al. [18]. This method is adapted and modified from an earlier TaqMan protocol by Schwaiger and Cassinotti [19], with a reported analytical sensitivity of approximately ten copies of TBEV RNA.

## Results

### ELISA

The number of positive and negative results in patients and controls is shown in Table 1. Borderline results were interpreted as negative. Among the 111 patients with serum samples available, only two lacked detectable IgM with either Enzygnost or Immunozym. One of these samples was positive in TBEV RT-PCR. According to the ECDC definition mentioned above [11], 117 of the reported 129 TBE cases (90.7%) could be confirmed retrospectively. Of the 12 cases that could not be confirmed, one case had only one serum sample, drawn on day 10, which was IgM positive but IgG negative. One case lacked detectable TBEV antibodies both in serum and CSF. For the remaining ten cases, only CSF samples could be located in the laboratory. All of these were IgM negative and eight of ten were IgG positive. (See Supplementary table).

**Table 1:** Number of positive (+) and negative (−) results of 248 patient samples from serum and CSF, 140 control serum samples and 10 CSF control samples. Borderline results were interpreted as negative

Sample	TBEV ELISA test	Positive (+)	Negative (–)
Patient serum (*n* = 153)	Immunozym IgM	122 (79.7%)	31 (20.3%)
	Enzygnost IgM	134 (87.6%)	19 (12.4%)
	Immunozym IgG	141 (92.2%)	12 (7.8%)
	Enzygnost IgG	138 (90.2%)	15 (9.8%)
Patient CSF (*n* = 95)	Immunozym IgM	30 (31.6%)	65 (68.4%)
	Immunozym IgG	84 (88.4%)	11 (11.6%)
Serum controls (*n* = 140)	Immunozym IgM	1 (0.7%)^a^	139 (99.3%)
	Enzygnost IgM	1 (0.7%)^a^	139 (99.3%)
	Immunozym IgG	41 (29.3%)	99 (70.7%)
	Enzygnost IgG	34 (24.3%)	106 (75.7%)
CSF controls (*n* = 10)	Immunozym IgM	0	10 (100%)
	Immunozym IgG	0	10 (100%)

^a^Samples from the same blood donor

#### Serum samples

Two hundred ninety-three serum samples, 153 from 111 TBE-patients and 140 from blood donors, were analyzed with both Immunozym and Enzygnost for TBEV antibodies.

##### Patient samples

Positive test results were obtained for TBEV-specific IgM in 98.2% (109/111), and 97.3% (108/111) of the patients had detectable TBEV specific IgG antibodies when compiling the results from one or both of the ELISA tests (Table 2).

**Table 2:** Number and percentage (%) of 129 patients notified with TBE in Western Gotaland, Sweden, during the years 2004–2012, with positive test results regarding TBEV-specific antibodies in serum and cerebrospinal fluid (CSF)

Test	Serum (*n* = 111)	CSF (*n* = 88)
Immunozym IgM	109 (98.2%)	29 (33.0%)
Immunozym IgG	108 (97.3%)	81 (92.0%)
Enzygnost IgM	109 (98.2%)	
Enzygnost IgG	106 (95.5%)	

##### Blood donor controls

One person was positive for both IgM and IgG in both ELISA tests. This was considered indicative of an ongoing or recent TBEV infection, which could pose a risk for viral transmission by transfusion. However, RT-PCR for TBEV RNA on the same sample was negative. Since the blood donors were anonymous, no further information about that specific case could be obtained. Of the blood donors tested for TBEV-specific IgM, 139/140 (99.3%) were negative with both Immunozym and Enzygnost assays. For IgG, the Immunozym test yielded a positive result in 41/140 (29.3%) samples, as compared to 34/140 (24.3%) positive samples with the Enzygnost assay.

#### CSF samples

##### Patient samples

In total, 105 CSF samples (95 samples from 88 patients and ten samples from healthy controls) were analyzed for TBEV antibodies with Immunozym. In the notified patients, only 33% (29/88) were IgM positive in their CSF, while TBEV-specific IgG was detected in 92% of the patients (81/88) (Tables 2 and 3). In the first available CSF sample for each patient, 88.6% (78/88) of the patients had detectable TBEV-specific IgG.

**Table 3:** Number and percentage (%) of patients with different TBEV serology results in serum (s-) and CSF (sp-). A patient with at least one positive serology result is considered positive

Serology results serum samples	s-IgM + s-IgG –	s-IgM + s-IgG +	s-IgM – s-IgG +	s-IgM – s-IgG –
Total, *n* = 111	1 (0.9%)	108 (97.3%)	0	2 (1.8%)
Serology results CSF samples	sp-IgM + sp-IgG –	sp-IgM + sp-IgG +	sp-IgM – sp-IgG +	sp-IgM – sp-IgG –
Total, *n* = 88	0	29 (33.0%)	52 (59.1%)	7 (8.0%)

##### Healthy controls

All of the ten control CSF samples were negative for both TBEV-specific IgM and IgG (Table 1).

### RT-PCR

Out of 126 blood samples analyzed, nine samples from eight patients were RT-PCR positive for TBEV RNA: eight samples from serum and one from whole blood. One of these samples (see Table 4) was sequenced, which confirmed TBEV of the European subtype (data not shown). All of the samples from CSF (*n* = 102), feces (*n* = 2) and nasopharynx (*n* = 5) were RT-PCR negative for TBEV. We were able to receive written informed consent to study the medical records from seven out of eight of the patients with positive PCR results. Of those seven PCR positive patients, 2/7 (29%) had a mild and 5/7 (71%) a moderate disease. The severity of disease was determined according to our previous study [20]. Mild disease was defined as primarily meningeal symptoms with the following symptoms: fever, headache, nausea, vomiting, neck stiffness, sensitivity to light and sound with a normal EEG or EEG not performed. Moderate disease was defined as moderate signs of encephalitis without or with slightly altered consciousness, and/or diffuse neurological symptoms such as confusion, slow thinking, or focal neurological symptoms such as ataxia, tremor and dysphasia. Severe disease was defined as multifocal symptoms and/or severe signs of encephalitis with altered consciousness.

**Table 4:** CT values and description of the eight RT-PCR positive patients, in relation to Enzygnost serology findings

Patient number	Age (years)	Gender (male/ female)	PCR positive day after fever onset	CT value in duplicate	Sample type	Disease severity	Duration of hospital stay (days)	Serology result IgM (Enzygnost)	Serology result IgG (Enzygnost)
1	70	F	5	34.0/33.5	Serum	Moderate	15	**–**	**–**
2	35	M	6	34.9/35.7	Serum	Unknown	Unknown	**+**	**–**
3	66	F	2	36.1/35.3	Serum	Mild	27	**–**	**–**
4	14	F	8	37.1/35.8	Serum	Mild	15	**+**	**–**
4			8	37.0/37.8	Whole blood				
5	46	M	10	39.5/38.5	Serum	Moderate	12	**–**	**–**
6	27	F	2	32.0/31.2^a^	Serum	Moderate	20	**+**	**–**
7	41	F	7	34.2/34.0	Serum	Moderate	3	**+**	**–**
8	54	F	15	29.1/29.3	Serum	Moderate	9	**–**	**–**

^a^Sequencing of TBE virus (7 kb) was performed and confirmed the European subtype

The median age of the eight RT-PCR positive patients was 43.5 years, with a range of 14–70 years. In six of seven patients, a biphasic disease was recorded while one had a monophasic course. The positive real-time RT-PCR result was detected on median day 6 after fever onset (range 2–15), with a mean CT value of 34.4 (range 29.1–39.5) (Table 4). One patient, a 54-year-old woman with a moderate encephalomyelitis, had a positive RT-PCR early in the second phase of the disease, before TBEV-specific IgM and IgG antibodies could be detected. The seven RT-PCR-positive patients were hospitalized for between 3 and 27 days, with a median of 15 days. There were no major concomitant diseases documented, and none of these cases was considered to be immunosuppressed. Four of the eight PCR positive patients had detectable IgM in serum but all of the eight patients were IgG negative at the time when the RT-PCR positive sample was drawn (Table 4).

## Discussion

TBE is a notifiable disease in Sweden and a high diagnostic standard is of utter importance. Our re-evaluation of samples from notified TBE patients showed a high diagnostic accuracy regarding the serology, where only serum from two of the notified patients lacked TBEV-IgM, and where 117/129 of the reported TBE cases could be retrospectively confirmed according to the current ECDC definition.

Previous studies [21, 22] have underlined the importance of cross-reaction between antibodies to different flaviviruses. Unlike IgG, IgM antibodies against other flaviviruses are rarely detected in TBE [23], and if they do occur, the titers are usually low [24]. Stiasny et al. [24] therefore suggested additional testing of all patients with low positive results to confirm the TBE diagnosis and to avoid the risk of false positive results caused by persisting IgM antibodies, vaccine-induced IgM or recent or ongoing other flavivirus infections. However, except for one blood donor with a possible ongoing TBE infection (but PCR-negative), all of the 206 serum control samples in our study were TBEV IgM negative. In a clinical setting other than for our regional suspected TBE cases, such as when diagnosing fever among travelers, the antibody cross-reactivity with other flavivirus infections and/or previous vaccinations may play a more important role [21].

CSF antibody testing for TBEV-specific IgG proved to be of great diagnostic value, since 88.6% of the patients were positive in their first drawn sample and this analysis could therefore be considered to be included among other diagnostic criteria. In contrast, only a third of the here reported cases fulfilled the ECDC criteria of IgM-positivity in CSF, an outcome that should be further studied. Our study detected TBEV IgM antibodies in 33% and IgG in 92% of the cases, using Immunozym, which is the only commercial assay available for CSF analysis in Sweden. Thus, despite the fact that the first drawn CSF sample from each patient was analyzed, IgG analysis was of greater value than was IgM. The reason behind this seemingly rapid IgG response in the CSF is unclear, but kinetic studies could be of interest.

Concerning the here unconfirmed patients, based on our detection of CSF IgG antibodies, it is probable that an additional 9/12 subjects were actual TBE cases, but could not be confirmed due to lack of serum samples. The results of the present study underscores results from previous studies [14], that in early cases of TBE, which lack IgG and sometimes IgM, this infection can be readily diagnosed by RT-PCR analysis of serum. By additional analysis of TBEV-specific IgG in the CSF, cases can be diagnosed, where CSF IgM may be absent and only IgG remains in the CSF.

RT-PCR for TBEV was positive in nine blood samples from eight patients. All of these were drawn at an early stage of the disease, although one in the second disease phase. None of the RT-PCR positive samples had a detectable TBEV-specific IgG, whereas four were IgM positive, suggesting a clearance of TBEV from the circulation as soon as the IgG antibodies are produced. This is in line with earlier findings [14]. Saksida et al. found PCR positive samples in 30/30 Slovenian patients presenting with fever after a recent tick bite, before TBEV-specific antibodies appeared. After development of TBEV IgM antibodies, 3/13 were RT-PCR positive, and only one sample was RT-PCR positive after IgG seroconversion [15]. Our study confirms previous findings and we conclude that RT-PCR in serum can be useful as an early diagnostic tool and as a means of collecting TBEV RNA for sequencing and epidemiological studies.

Here, over 100 CSF samples were analyzed with RT-PCR for TBEV, a large number of samples compared to previous RT-PCR studies on this body fluid [14, 15], and all were negative. Since almost all of the investigated patients were IgG positive in the CSF, we suggest that the virus had already been cleared from this body fluid at the time of sampling. Our results confirm findings from previous studies [8, 14] and further underscore that RT-PCR on CSF samples does not substantially contribute to diagnosis of TBEV of the European subtype.

Our work points out problems with diagnosing TBE early in the disease when the serology is negative. At this stage, RT-PCR for TBEV performed on blood can be valuable and provide a diagnosis. We also show that analysis for TBEV-specific IgG antibodies, here detected in the CSF in a vast majority of the patients, is a valuable diagnostic asset that could be included in the clinical routine and in the ECDC diagnostic criteria when IgM antibodies are lacking in this body fluid.

## Electronic supplementary material

ESM 1 (DOCX 63 kb)
