# Supplementary material for: Diagnosing tick-borne encephalitis: a re-evaluation of notified cases
Source: Eur J Clin Microbiol Infect Dis. 2017 Nov 29;37(2):339–44. doi: 10.1007/s10096-017-3139-9 (PMC5780526; doi:10.1007/s10096-017-3139-9)
Supplement: Supplementary file 1 — (DOCX 63 kb) [file 10096_2017_3139_MOESM1_ESM.docx]

Supplementary table.

Description of general characteristics and TBEV ELISA results in the 12/129 cases that could not be confirmed according to the ECDC diagnostic criteria for TBE. In 10/12 of these patients no serum sample was available. Borderline results were interpreted as negative.

| **Gender** | **Age (years)** | **Sample day after disease onset** | **Material** | **Result Immunozym** |
| --- | --- | --- | --- | --- |
| M | 42 | 38 | CSF | sp-IgM -  sp-IgG + |
| M | 9 | 26 | CSF | sp-IgM -  sp-IgG + |
| M | 36 | 2 | CSF | sp-IgM -  sp-IgG - |
| M | 68 | 0 | CSF | sp-IgM -  sp-IgG - |
| M | 30 | 15  21  37 | Serum  CSF  Serum  Serum | s-IgM -  s-IgG -  sp-IgM -  sp-IgG -  s-IgM -  s-IgG -  s-IgM -  s-IgG - |
| F | 47 | 29 | CSF | sp-IgM -  sp-IgG + |
| F | 55 | 23 | CSF | sp-IgM -  sp-IgG + |
| M | 42 | 10 | Serum | s-IgM +  s-IgG - |
| F | 67 | 3 | CSF | sp-IgM -  sp-IgG + |
| M | 36 | unknown | CSF | sp-IgM -  sp-IgG + |
| K | 62 | 9 | CSF | sp-IgM -  sp-IgG + |
| K | 58 | 21 | CSF | sp-IgM -  sp-IgG + |
